# A Review of Bio-Inspired Perching Mechanisms for Flapping-Wing Robots

**DOI:** 10.3390/biomimetics10100666

**Published:** 2025-10-02

**Authors:** Costanza Speciale, Silvia Milana, Antonio Carcaterra, Antonio Concilio

**Affiliations:** 1 Department of Mechanical and Aerospace Engineering, Sapienza University of Rome, 00184 Rome, Italy; costanza.speciale@uniroma1.it (C.S.); silvia.milana@uniroma1.it (S.M.); antonio.carcaterra@uniroma1.it (A.C.); 2 Special Projects Unit, The Italian Aerospace Research Centre (CIRA), 81043 Capua, Italy

**Keywords:** flapping-wing robots, ornithopters, perching mechanisms, grasping, robotics

## Abstract

Flapping-Wing Aerial Vehicles (FWAVs), which take inspiration from the flight of birds and insects, have gained increasing attention over the past decades due to advantages such as low noise, biomimicry and safety, enabled by the absence of propellers. These features make them particularly suitable for applications in natural environments and operations near humans. However, their complexity introduces significant challenges, including difficulties in take-off and landing as well as limited endurance. Perching represents a promising solution to address these limitations. By equipping these drones with a perching mechanism, they could land on branches to save energy and later exploit the altitude to resume flight without requiring human intervention. Specifically, this review focuses on perching mechanisms based on grasping. It presents designs developed for flapping-wing platforms and complements them with systems originally intended for other types of aerial robots, evaluating their applicability to FWAV applications. The purpose of this work is to provide a structured overview of the existing strategies to support the development of new, effective solutions that could enhance the use of FWAVs in real-world applications.

## 1. Introduction

The morphing wing is a research topic that, in spite of its ancient roots [1], has been developing significantly in the last 25 years, starting from reference research conducted by Jay Kudva [2], within the adaptive wing program. However, after many experiments, pieces of research and realization, culminating in the 2015 NASA experiment [3], actual applications of morphing aerodynamic surfaces on commercial aircraft seems too far away to be reached with the current state of technology. As in many other branches of engineering, changing scale is the most obvious trial to shorten the implementation time on real objects. Jay Kudva started their own enterprises based on the development of next generation aircraft, focusing on adaptive wing architectures [4]. In a fast sequence, studies were applied to insect [5,6] and bird-like [7] flying machines.

The surprising result of the first experiences was that those mechanisms were incredibly robust, in the sense that simple machines were able to fly. The focus was then placed on the implementation of certain maneuvers, particularly take-off and landing, as well as the optimization of flight performance. Many devices are available on the market, even remotely controlled ones [8,9,10], but there is a long way to go before the realization of autonomous systems with sufficient autonomy and range performances.

Flapping-Wing Aerial Vehicles (FWAVs), also known as ornithopters, can be said to arise from the desire of researchers to develop Unmanned Aerial Vehicles (UAVs) that replicate the unique flight capabilities of natural fliers, refined through millions of years of evolution. Like birds and insects, these robots generate both the lift and thrust necessary to move forward and sustain their weight and even some payload, solely through the flapping of their wings. These objects have unique properties, making them engineering realizations of wide applicative interest.

The absence of noisy rotating propellers, combined with their morphological resemblance to natural fliers, makes them ideal instruments for operations such as environmental monitoring and sample collection in sensitive ecosystems, where minimal disturbance is essential. In fact, the generated noise possibly falls in the spectrum of the typical inhabitants of the specific target environment, and its impact could be considered minimal, in principle. They can be used as “natural” monitors for several applications, such as wildlife protection, fire prevention and assistance to firefighters and civil protection, intelligence, and so on. Associated with a great potential, these platforms face significant challenges for real-world implementation. For instance, strict weight constraints limit onboard energy storage, and therefore endurance, and reduce take-off and landing capabilities.

From the optimization of the flight itself, to the arrangement of integrated devices making the system able to accomplish different flight phases, or the improvement of velocity and range of velocity, to the deployment of effective systems for take-off and landing, these objects may be predicted to envisage many improvements in the years to come. In this paper, the attention is focused on perching, which is, for a common bird, the phase of flight stand-by, as it stops on a branch, a pole, electrical wire or whatever grants it a proper purchase.

Perching is a promising solution to overcome some of the mentioned limitations that has gained increasing attention in recent years. There are many advantages associated with this enforced capability. For instance, by allowing flapping-wing robots to rest on branches through the use of dedicated perching mechanisms, mission duration may be extended in many ways. For example, if the robot is supported by solar power, its batteries can be recharged. Under non-optimal conditions (at night), it can rest safely somewhere. The ability to stop at altitude, especially for large systems, can ensure repeated overflights since, in favorable environmental conditions, as in the case of wind presence, the mechanical aircraft can return to its base (or another point) at height without a large consumption of stored energy, bypassing the crucial and, currently, severely limiting phase of the take-off. While perched, these platforms can continue operational tasks such as monitoring the surrounding region.

Various perching strategies exist. Vehicles can perch either by grasping the surface with a mechanical gripper or by attaching to it by several bonding techniques (e.g., by adhesives, magnets or even vacuum pumps) [11]. Typically, these latter techniques impose constraints on the landing surface properties, making them less suitable for natural environments according to the opinion of the authors.

This work focuses on grasping-based perching mechanisms, which, according to the available literature, are still in their early stages of development for FWAVs. The first contribution in this field is relatively recent and corresponds to the Shape Memory Alloys (SMA)-actuated system developed by Gomez-Tamm et al. [12] for a 450 g ornithopter. This design enabled the grasping of perches and objects but required remote operator control for opening and closing and was not tested in a complete perching maneuver. Since then, interest in grasping-based perching mechanisms for flapping-wing robots has grown, leading to several advancements, including the first autonomous perching flight of an ornithopter achieved by Zufferey et al. [13]. More recent contributions include that by Broers et al. [14], who designed a Fin-Ray-based perching mechanism for a FWAV with particularly limited payload capacity, and Hammad et al. [15], who employed SMA actuators to develop a lightweight mechanism scalable to flapping-wing robots of different sizes. Despite these advancements, as will be discussed, several critical challenges remain, and many opportunities for further technological progress still exist.

To support the development of new and effective devices for flapping-wing platforms, this review also includes perching systems designed for other aerial vehicles, such as multirotor and fixed-wing drones. Notably, research on bird-inspired perching for UAVs first emerged in the context of multirotors, with early contributions, such as that of Doyle et al. [16], published nearly a decade before the first FWAV-specific work. Since then, a wide variety of prototypes have been developed, providing valuable insights for designing systems tailored to flapping-wing robots.

The mechanisms reviewed are discussed in terms of their suitability for flapping-wing platforms, which exhibit sufficient payload capacity to integrate them onboard. According to the classification proposed by Nekoo et al. [17], these include primarily large-scale (over 100 g) and some small-scale systems (1–100 g). Referring instead to the classification by Hammad et al. [18], the focus lies on bird-like and hybrid FWAVs. In addition to payload capacity, an important difference between platforms of different sizes is their flight behavior. Smaller robots, which typically flap their wings at higher frequencies, generally possess hovering and Vertical Take-Off and Landing (VTOL) capabilities. As robot size increases, flapping frequency, and with it the ability to hover, tends to decrease. In fact, larger platforms generally require significant forward velocity to generate appropriate lift and are therefore limited to forward flight and gliding. As will be discussed, these differing behaviors strongly influence the design requirements of perching mechanisms.

This review adds to previous valuable contributions that have already addressed the topic of perching in aerial robots. Meng et al. [11] provided an overview of UAVs equipped with grasping, manipulation and perching capabilities. Their focus lies mainly on multirotor platforms; since its publication, several more recent studies have become available. Hammad et al. [18] analyzed landing and take-off strategies across all types of FWAVs, with perching mechanisms included as part of that broader topic. Significant insights in general mechanisms of flapping-wing robots may be found in recent reviews, such as the ones by Song et al. [19] and Ma et al. [20], focusing on the design of insect-inspired and bird-like FWAVs, respectively. Zhang et al. [21] reviewed platforms with deformable wings, while Han et al. [22] examined flapping mechanisms for bird-inspired platforms, and Fang et al. [23] addressed flight control methods. Finally, a broader overview of recent progress and challenges in design, modeling and control of flapping-wing robots is provided by Nekoo et al. [17].

In this context, the present review offers a dedicated analysis of grasping-based perching mechanisms for aerial robots and discusses their suitability for proper installation on flapping-wing platforms, with the aim of providing an assessed scenario for further supporting their development. The review is also considered useful to researchers interested in setting up perching devices for other types of aerial vehicles, as it is also intended as a tool for merging and exploiting the different concepts to other scenarios.

The review is organized as follows: Section 2 introduces the different classification criteria of perching mechanisms, based on the main system characteristics; it is mainly intended to provide a structured way to properly approach different existing designs. Section 3 explores different grasping-based perching mechanisms proposed over the years, classifying them into those specifically designed for flapping-wing or other types of flying robots; such an analysis allows several insights into their engineering. Section 4 offers a comparative discussion of design characteristics and assesses the apparent applicability of the different solutions to FWAV platforms. Finally, Section 5 concludes the review and highlights potential directions for future research.

## 2. Classification of Perching Mechanisms for Aerial Robots

**Figure 1:**
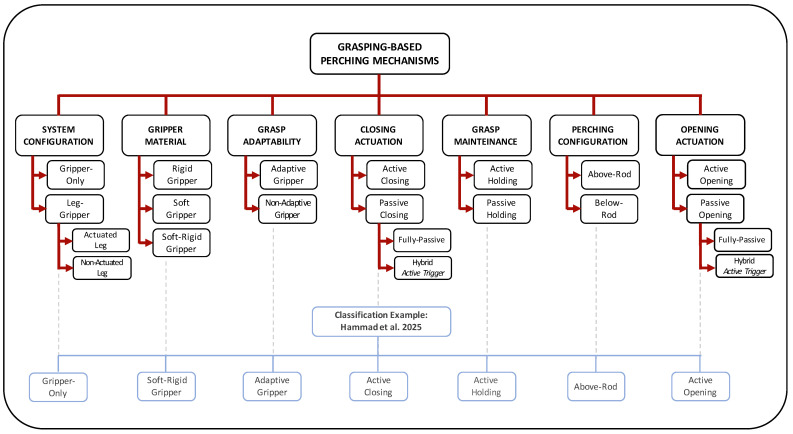
Classification criteria for grasping-based perching mechanisms, with an example of classification (Ref. [15]).

In this section, different classification methods for grasping-based perching systems are presented, based on key system characteristics. These are summarized in the diagram reported in Figure 1. Each group corresponds to a distinct and independent classification criterion, addressing a different aspect of the perching mechanism. When considered together, these criteria can help gain a deeper understanding of how each system works, its strengths and limitations and the cases in which it may be most effective. This approach is believed to be particularly valuable for guiding the selection of optimal solutions for FWAV applications.

### 2.1. System Configuration

Some grasping-based perching mechanisms [16,24] consist of a gripper mounted directly on the drone frame, while others include an intermediate leg between the drone and the gripper [25,26].

**Figure 2:**
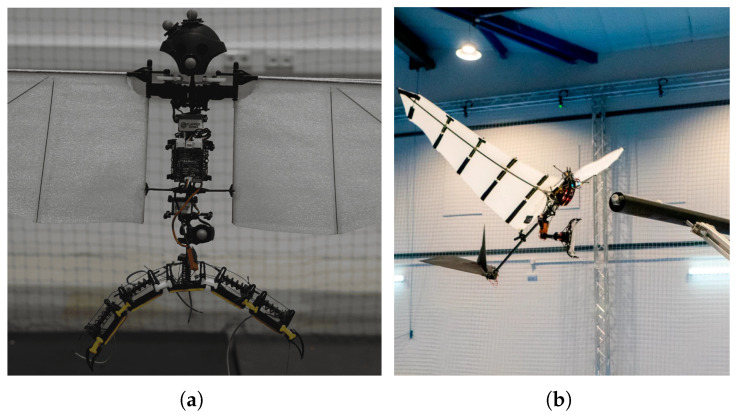
(**a**) Example of a Gripper-Only Perching Mechanism. Adapted from [15], licensed under CC BY 4.0. (**b**) Example of a Leg-Gripper Perching Mechanism. Adapted from [13], licensed under CC BY 4.0.

While a gripper-only design (Figure 2a) offers advantages in terms of weight, compactness and simplicity of actuation and control, adding a leg can introduce important benefits, particularly when it is actuated. An actuated leg, like the one shown in Figure 2b, allows for control over the gripper’s position and orientation and thus can compensate for positional errors during landing and also help stabilize the system over the perch. Non-actuated legs can still provide some advantages, including decoupling the gripper from the drone frame and helping absorb impact forces, thereby reducing the risk of damage to the robot. Moreover, as will be explained, in some cases such a leg can be used to achieve passive closure of the gripper during landing. However, the drawbacks of adding a leg should also be considered. In addition to increased weight and bulk, there is also the potential for flight instability due to asymmetry or swinging if the mechanism is not properly retracted or restrained.

### 2.2. Gripper Material

According to the stiffness of the gripper material, grippers can be divided into rigid, soft and soft-rigid types [27].

Rigid grippers are made of stiff materials such as metals, hard plastics or composites. These are generally able to provide high gripping forces, accompanied by a high level of repeatability.

Soft grippers, instead, are made from flexible materials like silicone elastomers and Thermoplastic Polyurethanes (TPUs). These offer great adaptability to different object surfaces and are safer for the manipulation of delicate objects, which is particularly important if the gripper is intended to be used not only for perching. However, due to their inherent low stiffness, soft grippers generally cannot generate adequate gripping forces. To address this limitation, a common solution is to combine flexible and stiff materials in a soft-rigid gripper, in order to merge the advantages of both approaches.

### 2.3. Grasp Adaptability

Another way to classify grippers is by their ability to conform to the shapes of the objects they grasp.

**Figure 3:**
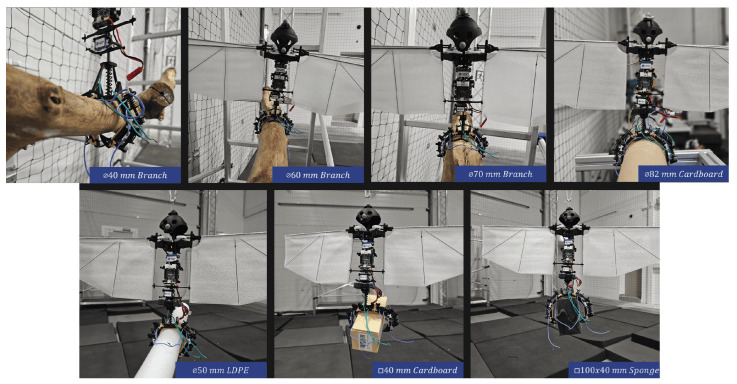
Example of an Adaptive Gripper grasping different objects. Adapted from [15], licensed under CC BY 4.0.

Adaptive grippers change their shape to match the object, as shown in Figure 3, providing high versatility and enabling them to handle a wide range of perch geometries. This adaptability is typically achieved through underactuation and/or material compliance [11], as will be discussed.

In contrast, non-adaptive grippers maintain a fixed geometry. While they are mechanically simpler and generally faster to actuate, they tend to provide less contact area, which often results in a less stable grasp. These grippers are often used in scenarios where the properties of the object to be grasped are well-known, allowing for optimization to fit the particular shape. However, they are less suited for applications such as perching in natural environments, where the size and shape of branches can vary significantly.

### 2.4. Closing Actuation

Referring to the closing phase, perching mechanisms can be divided into two main categories: passive and active closing systems.

Passive closing systems do not rely on powered actuators to perform closure but instead harness mechanical energy. This energy may come, for instance, from potential energy stored in springs [13] or flexible elements [28], which drives the closure when released, or from the kinetic energy of the robot, which, at the impact, can trigger mechanisms such as bistable structures to switch from an open to a closed state [29]. These systems are typically lightweight and offer a fast response, but they lack the ability to modulate grip force and may feature a slow reset to return to their initial state. This category can be further divided based on whether or not active components are involved in the process between receiving the closure command and executing the closure. Two main types can be identified: passive closing systems with active trigger, which can also be referred to as hybrid systems, where the closure itself is passive but requires an active trigger to initiate the motion (e.g., a system where a servo is used to release a latch), and fully passive closing systems, in which no active components are involved at any stage.

Active closing systems, on the other hand, employ actuators, such as electric [30] or pneumatic ones [25], to directly drive the closing motion. These systems are preferable when precise control over grip force or timing is required. However, given the payload limitations of aerial robots, the choice of actuators is generally restricted, limiting the closure speed achievable by an active closing gripper.

### 2.5. Grasp Maintenance

Once the closure is completed, the gripper must maintain its closed configuration to keep the robot anchored to the perch for as long as needed.

Most existing grasping-based perching mechanisms are designed to maintain the grasp passively, requiring no energy input once the gripper is closed. These are referred to as passive holding mechanisms. As will be discussed, various strategies have been proposed to enable passive grasp maintenance, including bistable structures [31], mechanical latches [32], locking ratchets [33] and configurations that rely on the vehicle’s own weight to keep the gripper closed [16].

However, some systems do require a continuous or intermittent energy input to maintain the grasp. Although their energy demands are typically low, this limitation can reduce the duration of perching, making passive holding mechanisms generally preferable.

### 2.6. Perching Configuration

Based on the posture of the robot after perching, mechanisms can be classified as achieving either an above-rod or below-rod configuration.

**Figure 4:**
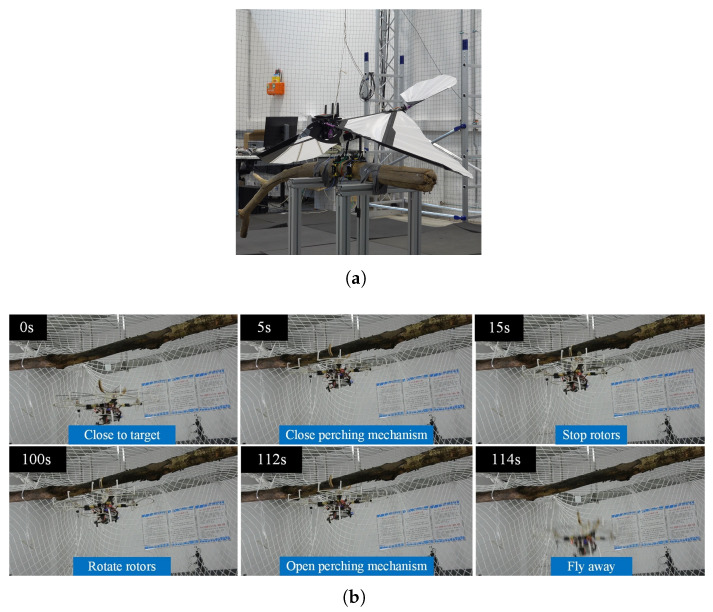
(**a**) Example of an Above-Rod Perching Mechanism. Reproduced from [15], licensed under CC BY 4.0. (**b**) Example of a Below-Rod Perching Mechanism mounted on the upper part of the drone frame. Adapted from [34], licensed under CC BY 4.0.

In the first configuration (Figure 4a), the gripper must generate enough gripping force to firmly hold the branch through static friction, preventing slippage. For this reason, mechanisms in this category often incorporate finger pads made from materials specifically selected to increase friction at the gripper–perch interface. Another important factor in preventing slippage is positioning the robot’s center of mass as close as possible to the vertical axis passing through the branch, thereby minimizing the gravity-induced moment. Thus, typically, having an actuated leg that can adjust the robot’s position to achieve this alignment is advantageous.

In the second configuration, the drone hangs below the branch, either because the perching mechanism is mounted on the upper part of the frame (requiring the vehicle to approach from below, as shown in Figure 4b), or because the gripping force provided is insufficient to hold the branch, resulting in slippage. These systems are generally designed for multirotor platforms, which can take off even from an upside-down configuration. In such cases, take-off is typically achieved by activating the propellers while the gripper remains closed, allowing the drone to rotate upright before releasing the perch and flying away. For robots like FWAVs, however, such a maneuver would be significantly more challenging, making this type of system generally unsuitable for their use.

### 2.7. Opening Actuation

Similar to the closing phase, grippers can also be categorized based on the type of actuation used for opening.

Active opening systems use actuators to drive the fingers back to the open configuration. These include not only grippers that actively close and then use the same actuator to reverse the motion [26] but also those with passive closure that require active actuation to reopen [33]. Many of these systems rely, for example, on energy stored in passive elements, such as springs, for closure and typically require an actuator to reload them in preparation for the next grasp.

There are also examples of passive opening systems, which, similar to the classification defined for closure, can be further divided into fully-passive systems [16] and hybrid ones [32]. In the latter, an actuator is required to perform an intermediate action such as disengaging a locking mechanism, after which the gripper passively returns to the open state.

## 3. Perching Mechanisms

In this section, various grasping-based perching mechanisms developed for aerial robots are presented. Although the primary focus of this review is on systems for flapping-wing drones, the number of such mechanisms remains relatively limited. Solutions for the perching of other aerial platforms, such as multirotor and fixed-wing UAVs, have been more extensively explored. Including these designs in the analysis is therefore considered highly valuable, as they can offer important references for developing effective mechanisms for flapping-wing robots. Accordingly, the section is divided into two main parts: the first analyzes grasping-based perching mechanisms developed and tested on flapping-wing robots, while the second focuses on those designed for other types of aerial vehicles. To maintain readability and avoid overwhelming the reader, the mechanisms will not be exhaustively categorized according to all the criteria introduced in Section 2. Instead, only the most relevant and distinctive classification aspects will be mentioned where appropriate. Full classification details for each mechanism can be found in Table 1.

### 3.1. Perching Mechanisms for FWAVs

A notable early contribution to the development of perching mechanisms based on grasping for flapping-wing robots was made by Gomez-Tamm et al. [12], who developed a bio-inspired system composed of two claws mounted at the ends of the robot’s legs. Each claw has four fingers made of Polylactic Acid (PLA) phalanges connected by flexible Thermoplastic Polyurethane (TPU) joints. The claws are tendon-driven, with tendons routed internally through the fingers, resulting in a soft-rigid, underactuated gripper capable of adaptively grasping objects of various sizes and shapes. The closing and opening of the claws are remotely triggered and are achieved by alternately heating two SMA springs per leg, arranged in an antagonistic muscle configuration. While the closing motion is actively driven, this setup enables passive grasp maintenance, as the claws require no additional energy to remain closed. Although the system demonstrated the ability to grasp, hold, and release different objects, and even to support a 450 g ornithopter on a rod until (tethered) take-off, it is not capable of performing a full perching maneuver. One key limitation is the absence of an actuator at the hip joint to control leg orientation. This limitation was addressed in a successive and modified version of the system by Perez-Sanchez et al. [35], who introduced active position control of the leg via SMA actuators through a finite-time state-dependent differential Riccati equation control strategy.

**Figure 5:**
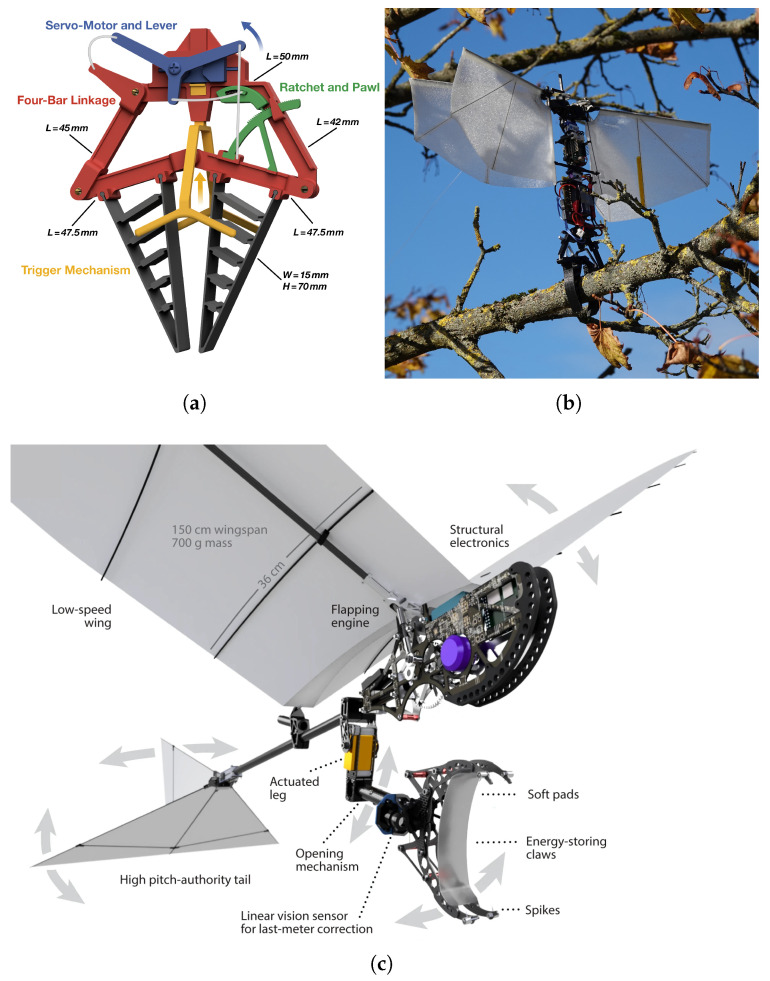
(**a**,**b**) Fin-Ray gripper design by Broers et al. Reproduced from [14], with permission of the authors. (**c**) Claw-leg system by Zufferey et al. Adapted from [13], licensed under CC BY 4.0.

Broers et al. [36] were the first to focus on grasping-based perching for small-scale, hover-capable flapping-wing robots. To this end, they proposed a soft, adaptive gripper-only mechanism based on the so-called Fin-Ray effect, a phenomenon observed in fish fins [37] and commonly used for the design of adaptive fingers for robotic grippers [38,39,40]. Thanks to their structure, consisting of two bones forming a V-shape with connective tissue in between, fish fins bend in the direction opposite to that of an applied force. This behavior is replicated in robotic fingers using a triangular frame with cross-struts connected by hinges, allowing them to conform and wrap around objects when pressed against them. The proposed gripper, shown in Figure 5a,b, integrates two Fin-Ray fingers and is actuated via a four-bar linkage mechanism driven by a servomotor, with opening and closing decoupled and achieved by pulling different bars. The active closure is triggered by a fork that, upon contacting the perch, activates a mechanical switch. Once closed, the gripper remains locked without energy expenditure thanks to a built-in ratchet mechanism. Although intended for a FWAV, the initial design was tested only on a quadrotor. A later version, which incorporated improvements such as a resettable ratchet for reopening and reduced weight [14], was mounted under a commercially available flapping wing-robot [10] and demonstrated successful perching in manual, free-fall and controlled flight conditions.

The first autonomous perching flight of a flapping-wing robot on a perch was demonstrated by Zufferey et al. [13]. They developed a claw-leg system that was mounted under a large-scale ornithopter weighting 700 g. Combined with a flapping-flight controller and a close-range correction system, this setup achieved a perching success rate of 66%, which was later increased to 88.3% [41] after some modifications. As can be seen in Figure 5, the perching appendage consists of a carbon-fiber leg actuated at the elbow joint, used to compensate for misalignment and maintain equilibrium once perching is completed, with a bistable claw mounted at its extremity. The claw is made of two rows of carbon-fiber plates with Ecoflex-covered toe pads, which are used to increase friction between the claw and the rod. The non-adaptive gripper is designed for branches with a diameter of 60 mm, with a decreasing gripping force as the branch diameter deviates from this value, limiting the system’s versatility. The mechanism automatically closes on the branch when it impacts two protrusions inside the claw in its open configuration. This impact causes the system to switch to the other stable configuration under the action of a spring, allowing for a fully-passive closing in 25 ms. Once closed, the claw remains locked thanks to its bistability, making it a passive grasp maintenance system. To reopen the claw, a tendon-pulling re-opening subsystem using a DC motor and leadscrew pulls the tendons, lifting the claws and returning the spring to its initial configuration. Despite the ability to reopen the gripper, the system does not enable take-off after perching.

To the authors’ knowledge, the most recent perching mechanism developed and tested on flapping-wing robots was designed by Hammad et al. [15]. It consists of two two-fingered soft-rigid underactuated grippers, each made up of multiple rigid phalanges connected by silicone joints and actuated by SMA springs acting on Kevlar tendons passing through them. The system can be categorized as an active closing and active opening mechanism. The closing of each gripper is achieved by applying current to expand a central spring, while opening occurs by supplying current to its antagonist springs located in each phalange. These antagonist springs, when compressed, draw the claw to the open position. An important limitation of this design is the need to supply energy to maintain the gripper’s hold over time, as the cooling of the springs causes them to lose gripping force. Several perching tests were conducted with the mechanism mounted under two different commercial platforms: a large-flapping wing robot [8] and a smaller VTOL-capable flapping-wing robot [10]. The perching tests with the first platform resulted in unsuccessful landings but successful tethered take-off, while both maneuvers were successfully completed with the second platform. This highlights how much more challenging the perching maneuver is for large-scale flapping-wing robots compared to those capable of hovering, which can approach the perch in a more manageable way for the perching system.

### 3.2. Perching Mechanisms for Other Aerial Robots

Research on grasping-based perching mechanisms for UAVs has so far primarily focused on multirotor platforms. This is largely due to their less stringent payload constraints compared to other aerial vehicles like FWAVs, as well as their VTOL and hovering capabilities, which allow them to approach and land on perches in a more controlled manner than other types of platforms, such as fixed-wing UAVs. As a result, the majority of the systems presented in this section have been developed and tested specifically for multirotor drones.

Mechanisms incorporating tendon-driven underactuated grippers, which represent a substantial portion of the solutions proposed to date, will be presented first. The analysis will then shift to other actuation strategies.

#### 3.2.1. Tendon-Driven Mechanisms

One of the most widely adopted solutions for developing a gripper for aerial robots’ perching is the use of tendon-driven underactuated mechanisms. The fingers of these grippers are typically composed of one or more rigid links (phalanges) connected by revolute joints, with inelastic cables (tendons) routed through them to enable simultaneous actuation of multiple joints. This approach significantly reduces weight compared to fully-actuated mechanisms, where each joint has a dedicated actuator. Another key advantage of tendon-driven mechanisms, particularly those with multiple phalanges per finger, is their inherent self-adaptability. By pulling or releasing the tendons (depending on the specific mechanism architecture, as will be discussed), the fingers close around the object and stop once each link comes into contact with the surface, passively conforming to its shape. The first contribution to the use of such grippers for perching was made by Doyle et al. [16], who developed a mechanism composed of a leg and a soft-rigid underactuated gripper. The leg consists of three rigid segments connected by pin joints, while the gripper has two toes, each with three phalanges connected by flexible joints. As the drone approaches the perch from above, its weight causes the leg to collapse, which in turn pulls a tendon routed through the leg and the gripper, closing the latter. The robot’s weight not only passively closes the gripper but also maintains the grasp over time without requiring energy input. Additionally, the removal of this load during take-off enables passive release of the grasp. To increase the adaptability, a pulley block is used to provide differential actuation for each toe, allowing them to conform to different perch geometries. The performances of the developed prototype were evaluated by mounting a pair of these leg-gripper mechanisms under a quadrotor and testing it on various common surfaces, including flat ones, and simulating a perching maneuver with a mock flight setup.

The use of a collapsing leg to pull the finger tendons and achieve a passive grasp is inspired by a mechanism once believed to enable passive grasping in birds, known as the Automatic Digital Flexor Mechanism (ADFM). By examining dissected specimens, researchers observed that, in some bird species, certain toe tendons were routed around the ankle and became tensioned when the leg bent, resulting in passive curling of the toes [42]. Another observation was that these birds had flexor tendons with small tubercles that matched the plicae in the adjacent tendon sheath, forming a ratchet-like tendon locking mechanism, known as the Digital Tendon-Locking Mechanism (DTLM) [43]. It was believed that, together with the ADFM, the DTLM enabled birds to maintain a grasp without any muscular effort, forming a mechanism collectively referred to as the avian Automatic Perching Mechanism (APM). However, Galton et al. [44], who conducted tests on live European starlings, demonstrated that these earlier assumptions were influenced by post-mortem tendon stiffness caused by rigor mortis in the examined specimens. Specifically, they observed that passive leg flexion did not produce toe flexion in anesthetized starlings and that the birds fell from their perches when unconscious. These findings indicate that these mechanisms are not sufficient for a bird to passively grasp and rest on a perch, but instead likely help reduce the muscular effort required to do so. Despite this, as will be discussed, several effective perching mechanisms have been developed that incorporate contact-triggered passive-closing or passive-holding features inspired by the ADFM and/or the DTLM, highlighting the potential of these biologically inspired strategies for developing efficient perching systems for aerial robots.

For instance, drawing inspiration from the ADFM, Nadan et al. [45] designed a dual leg-gripper mechanism that passively closes upon landing, thanks to a cable routed around the joints of each four-bar linkage leg and the phalanges of its corresponding multi-segmented, soft-rigid adaptive gripper. To enable perching from different approach angles, the legs can rotate, controlled by two servomotors located at the hip joints. Passive grasp maintenance is ensured against disturbances by a latch engaged by a servomotor, which holds the legs locked in the collapsed position. At take-off, the servo releases the latch, enabling a passive opening as the drone’s weight is removed. Tests conducted with the mechanism mounted beneath a hexa-copter demonstrated its ability to land, perch and take off from a wide variety of perching objects.

Another notable example, which integrates both the ADFM and the DTLM principles, is the system developed by Roderick et al. [33]. Their grasping-based perching mechanism, called SNAG (Stereotyped Nature-Inspired Aerial Grasper) consists of a pair of articulated legs with underactuated grippers. Each gripper can be categorized as soft-rigid and adaptive, as it comprises four fingers featuring rigid phalanges linked by elastic bands and covered with deformable toe pads enhanced with grip tape to improve friction. Servomotors located at the hips of the legs positions the feet toward the target and stabilizes the robot during landing. Upon impact with the perch, the legs start to collapse, pulling a tendon routed through them. These tendons are connected via a differential to additional tendons running into the toes, triggering a rapid grasp. A distinctive feature of this design is that the grasping force is further amplified by the simultaneous release of energy stored in a preloaded spring, also routed through the leg tendon. This release is enabled by a quick-release mechanism triggered by the leg’s collapse. As a result, the passive grasp is completed in under 50 ms. Then, a passive locking ratchet mechanism, in analogy with the DTLM, holds the gripper closed. At take-off, a motor resets the mechanism, reducing tension on the main tendon and allowing the fingers to passively open under the restoring force of the elastic bands at the joints, making it an example of a hybrid opening system. SNAG was mounted beneath a quadrotor and enabled both dynamic perching on natural branches and mid-air object grasping.

Wang et al. [46] also took inspiration from both avian mechanisms in designing their grasping and perching system. In this case, the closure of its tendon-driven, adaptive and rigid gripper is passively driven upon impact by a parallelogram leg. Noteworthy in their design is the locking mechanism, which passively maintain the grasp by directly replicating the morphology of a bird’s claw. Specifically, part of each finger’s tendon is surrounded by multiple conical ridge blocks that engage with grooves in a tendon sheath embedded in one of the phalanges. These structures mimic the function of tubercles and plicae in bird tendons and tendon sheaths, respectively. As a result, the mechanism works like a ratchet, allowing tendon movement only in the direction that curls the fingers, which occurs as the flexor tendon is stretched over the ankle joint as the leg folds. Releasing the perch is actively achieved by pulling an extension tendon via a motor. This tendon pushes the sheath downward, disengaging the locking mechanism and allowing the tendon to slide in the opposite direction, thus extending the fingers. Although the mechanism has not yet been integrated into a drone, several experiments were conducted to evaluate its performance. The claw allowed for successful grasping of cylindrical objects with diameters ranging from 10 to 40 mm, supporting loads of up to 9 kg, and performing stable perching from various approach angles.

A different approach was proposed by Firouzeh et al. [47], who developed a gripper-only mechanism in which closure is driven by the collapse of a Sarrus linkage whose motion is transmitted to the gripper via soft tendons. In this case, the gripper is non-adaptive, as each finger consists of a single claw with a fixed shape, limiting its effectiveness to a restricted range of perch geometries. A pair of these mechanisms was integrated into a quadrotor that performed perching maneuvers by approaching the perch from above, turning off its propellers and executing a free-fall onto the target. The gripper closes within 45 ms of impact and is then held closed by electro-adhesive clutches integrated into the tendons. Despite its low energy consumption, this active grasp maintenance method still limits perching duration. Another limitation is the system’s inability to exert an active squeezing force, which results in the drone settling into an equilibrium below the perch. For take-off, the claws are released passively by exploiting the elastic return of the tendons once the clutches are turned off.

In addition to these passive and bio-inspired mechanisms, actuators have also been directly employed to pull the tendons of underactuated grippers and drive their closure. Popek et al. [48] proposed an adaptation of the open-source Yale OpenHand Model T gripper [49] for the autonomous perching of a multirotor on cylindrical structures. The underactuated fingers of the soft-rigid gripper, each composed of two links connected by compliant pin joints, are driven simultaneously by a servomotor acting on a tendon routed through a pulley differential. The mechanism enabled the drone to achieve static perch angles up to approximately 30 and to passively maintain its grasp thanks to the low back-drivability of the designed transmission. More recently, a similarly active closing system was developed by Iida et al. [50]. In their design, the independent movement of each rigid finger was achieved using a specially designed pair of two-dimensional differential plates. These plates were connected on one side to a single active tendon driven by a servomotor, and on the other side to three flexor tendons and three extensor tendons, respectively. This configuration enabled the system to adaptively grasp a wide variety of objects and to perform multidirectional perching with a fully-actuated quadrotor.

An alternative strategy for driving the closure of tendon-driven underactuated grippers involves leveraging the restoring forces generated by elastic joints designed to maintain the closed state. By initially forcing the extension of the fingers by pulling the tendons routed inside them, elastic energy is stored in the joints. This energy can then be used to drive the gripper closure when needed. In this case, the closure is not achieved by pulling the tendon, as in previous examples, but by releasing it. Moreover, since the equilibrium state is the closed position, this configuration allows the gripper to passively maintain the grasp over time.

This approach was used by McLaren et al. [28], who developed a soft-rigid robotic hand consisting of three fingers with two phalanges connected by a flexure joint in polyurethane elastomer. The fingers are anchored to the hand base by a spring-loaded pin joint. As described, each finger is initially flexed, and by pulling a tendon energy is stored in the joints. When a infrared proximity sensor detects an object within the hand’s grasping range, a servomotor triggers a quick-release mechanism, allowing the accumulated elastic energy to be rapidly released. This active trigger enables the hand to passively grasp the object in less than 96 ms. This 551 g system, which exhibits a maximum payload of 56 N, was tested on a quadrotor and successfully demonstrated above-rod perching and subsequent take-off, made possible by active opening of the hand through tendon retraction via a servomotor.

**Figure 6:**
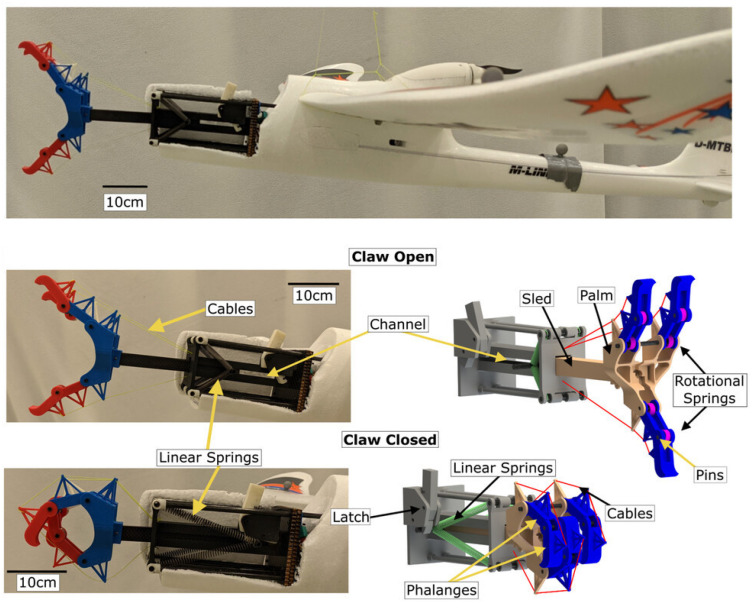
Perching Mechanism by Stewart et al., shown in both open and closed configurations. Adapted from [32], licensed under CC BY 4.0.

Stewart et al. [32] developed a similar gripper, also consisting of three fingers with two phalanges each, but equipped with rotational springs not only at the finger base, as in the previously described system, but also at the joints between the phalanges. A notable feature of their design is a mechanism that enables both passive closure and passive opening of the gripper by absorbing and storing kinetic energy during impact. This system, used for the perching of a fixed-wing UAV, includes a set of four linear springs connected on one side to the drone’s fuselage and on the other to a sled, which in turn is connected to the gripper palm. As illustrated in Figure 6, the sled is initially positioned fully forward, keeping the finger tendons under tension and the claws open. Upon impact with a perch, the sled slides backward, releasing the tendons and allowing the rotational springs to passively close the fingers. Simultaneously, the sled’s movement stretches the four linear springs, which are then held in place by a latch, allowing the gripper to passively maintain the grasp over time. Later, a servo disengages the latch to release the stored energy, reopening the claws for take-off. This perching mechanism proved effective in enabling the winged drone to perch on cylindrical rods across a yaw angle range of $58^{\circ }$, and at flight speeds ranging between 3.35 m/s and 7.4 m/s. It should be noted, however, that once closed, the gripper is not able to firmly hold the perch and slides, resulting in a below-rod perching configuration of the UAV.

More recently, Li et al. [26] designed a soft-rigid tendon-driven underactuated gripper that closes under the restoring forces exerted by its TPU-based joints in equilibrium in the closed state. However, here, the closing should be classified as active, as the release of the stored elastic energy in the joint is controlled by a servomotor directly acting on the tendons of the fingers, which results in a slower closing compared to passive systems. This mechanism was mounted on top of a quadrotor and successfully used for perching from below on three branches with diameters ranging from 30 to 80 mm.

#### 3.2.2. Other Perching Mechanism Designs

In addition to the widespread use of tendon-driven underactuated grippers, various alternative approaches have been explored over the years to design perching mechanisms for aerial robots. For instance, pre-stressed spring steel bands have shown good potential thanks to their rapid response upon contact and their intrinsic adaptability.

Nguyen et al. [29] proposed a perching system composed of preformed bistable spring steel metal encased in an inflatable fabric structure. By absorbing impact energy, this gripper is capable of transitioning from a straight to a curled configuration in about 4 ms, enabling passive grasping of a wide variety of objects. Once in its closed configuration, the grasper requires no additional energy to maintain the grasp, classifying it as a passive holding system. Conversely, opening is active, as grasp release is achieved by pneumatically inflating the fabric-based enclosure, which returns the bistable structure to its initial open state. Several successful perching experiments were conducted by free falling a soft-bodied quadrotor equipped with this grasper.

Spring steel bands were also used by Zengh et al. [31], who developed a perching mechanism compatible with standard rigid-bodied multirotors. Since such platforms tend to bounce when dropped onto a perch, an active triggering mechanism driven by a servomotor was introduced. Ultra-fast passive gripper closure (67 ms for the larger version and 42 ms for the smaller) is achieved by keeping two spring steel bands in a semi-stable open state, each held by a rigid bar applying force at the midsection. To initiate grasping, the servomotor rotates the bars, allowing the bands to rapidly snap into the closed state and grasp the perch. Once closed, the bistable nature of the gripper ensures a secure hold without energy consumption. Opening, however, requires energy as, to release the grasp, a second servomotor uncurls the bands by pulling strings routed through a system of gears and pulleys. The system was successfully tested outdoors on natural three branches, demonstrating reliable gripping without slippage on inclined surfaces.

As anticipated in the previous paragraph, another approach to achieving adaptive gripping is through the use of Fin-Ray fingers. In addition to the design already presented, developed for a FWAV, several perching mechanisms incorporating these fingers have been developed over the years for other aerial robots [51,52].

Among the most recent is the passive-closing and adaptive perching mechanism proposed by Askari et al. [24]. The passive closure of its claw, which features three adaptive fingers based on the Fin-Ray effect, is enabled by a Hoberman linkage leg that directs the robot’s weight to push the fingers to curl and wrap around the perch. Also, its opening is performed passively, since as the weight of the drone is progressively removed from the leg, the squeezing force decreases, allowing the claw to release the perch. Additionally, by reconfiguring the Hoberman linkage, the robot’s weight can be used to push the claw into a stretched configuration, enabling it to be used as a foot for walking. Several successful perching experiments were conducted with a 700 g quadrotor equipped with a pair of these leg-claw mechanisms, weighting 114 g in total. The gripper was also tested on perches with various shapes, including irregular ones and at different tilting angles, demonstrating good performance in conforming to the surfaces and preventing slipping.

Another recent design using Fin-Ray effect-based fingers was proposed by Lee et al. [53]. Developed for wood inspection through resistography, a technique involving drilling into wood to detect internal cavities, the system consists of four spring-loaded prismatic shafts and two grippers with two fingers, mounted on top of a quadrotor. As the drone approaches the wooden structure from below, the thrust from the propellers compresses the springs, causing the grippers to passively close around the structure. The holding phase in this setup should be classified as active, as the drone keeps its propellers active to hold the springs in compression and hover while drilling. Consequently, releasing is passively achieved simply by reducing the thrust, allowing the UAV to descend. Despite the system being designed for stabilizing the drone during overhead drilling, the prismatic gripper design could potentially be adapted for above-rod perching under the robot’s weight.

Ching et al. [25] explored an alternative approach for designing a soft and adaptive gripper for aerial robots perching, involving pneumatic actuation. They developed a soft actuator design, named the Film-Balloon (FiBa) module, which consist of a transversely curved polymer thin film and a 3D-printed pneumatic balloon that bends when inflated. These modules were used to construct a lightweight actively closing gripper with four fingers, each composed of two FiBa units, capable of adaptively grasping objects of various sizes and shapes. This gripper was mounted on top of a small-scale quadrotor weighting 249 g and was effectively used for perching from below on a branch, reliably supporting the system’s weight. One limitation is that, to maintain the grasp, the gripper requires continuous energy input to keep the pneumatic valves closed and to compensate the pressure loss over time.

**Figure 7:**
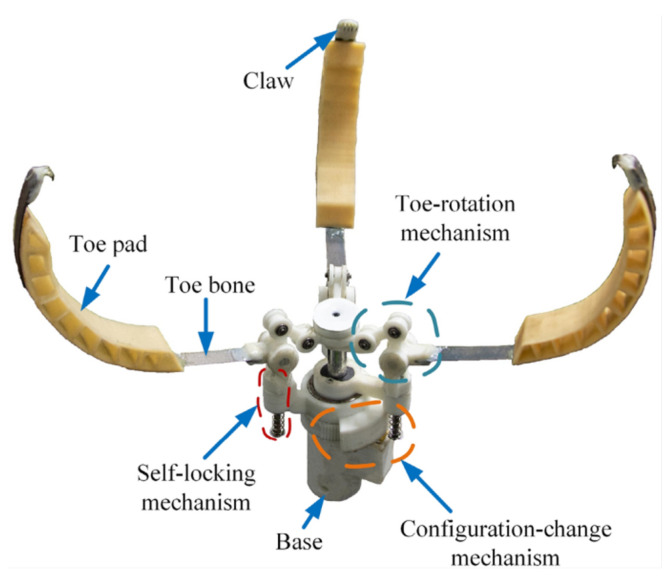
Perching mechanism by Bai et al. Reproduced from [34], licensed under CC BY 4.0.

Another active closing system was developed by Bai et al. [34]. Their soft-rigid gripper, shown in Figure 7, has three fingers, each composed of, by analogy with bird toes, a toe bone, a soft and deformable toe pad and a claw with multiple spines. This gripper adapts to the shapes of perching objects thanks to the porous structure of silica gel toe pads and the deformability of the toe bones made from spring steel. A notable feature of the design is that the fingers can switch between two configurations based on the shape of the object to grasp, thanks to a gear mechanism driven by a DC motor. Active closure and opening are performed under the action of a linear servo driving an inverted crank-slider mechanism. The system also incorporates a self-locking mechanism that helps save energy during perching. However, it should be classified as an active grasp maintenance system, as the DC motor remains powered during grasping. The mechanism was integrated onto the top of a quadrotor and enabled it to perch by hanging on a variety of objects.

Finally, moving to non-adaptive and non-tendon-driven perching mechanisms, several examples can be referenced [54,55].

Among them, the rigid gripper proposed by Hang et al. [30], composed of three carbon fiber fingers that actively close via three servomotors, coordinated to operate with a single degree of freedom. The gripper is mounted beneath a quadrotor and also includes contact modules specifically designed to match certain structures and allow the drone to rest on them. Closure is triggered by the controller once the drone reaches the designated perching position, after which equilibrium is reached with the drone resting upside down as the three fingers slide around the rod.

A non-adaptive gripper featuring passive closing was designed by Hsiao et al. [56]. Their system uses a bistable mechanism that enables the rigid gripper fingers to close automatically under impact forces during perching. When perching from below, the system transitions through three states. Initially, the gripper is in its open state. Upon contact, the impact force applied to a dedicated pad triggers a transition to the closed state, causing the fingers to grasp the perch. Once the drone’s propellers are turned off and no more force acts on the impact pad, the drone’s weight shifts the mechanism into a third, holding state. In this configuration, a latch passively maintains the fingers in the closed position, while the bistable mechanism resets to its open configuration in preparation for the next cycle. Opening is also passive: when the drone takes off and its weight is removed, the latch disengages, allowing two torsional springs to return the fingers to the open state. This fully passive system, weighting only 28 g, enabled a quadrotor to successfully perch and grasp objects and was capable of lifting loads up to 3.7 kg under static conditions.

Finally, to complete the overview of the presented systems, Table 2 complements Table 1, which reports their full classification, by providing values for several key parameters, enabling a comparison of performance and characteristics across designs. These parameters include, for example, the grasping force, intended here as the contact force exerted by the gripper on the object. Due to variations in experimental evaluation methods, slight differences in the meaning of the reported values may exist. Readers are therefore referred to the original articles for details. Other quantities included are the load capacity of the mechanism, intended as the force required to pull the gripper from the object grasped; the force-to-weight ratio, calculated as the ratio between the reported grasping force or load capacity and the perching mechanism weight; and the mass ratio, representing the percentage of the perching mechanism weight relative to the total UAV weight.

**Table 1:** Complete classification of the examined grasping-based perching mechanisms.

Ref.	Yr	Aerial Platform	System Config.	Material	Adaptive	Closing Actuation	Grasp Maint.	Perching Config.	Opening Actuation
[15]	2025	FWAV	Gripper-Only	Soft-Rigid	Y	Active	Active	Above-Rod	Active
[26]	2025	Quadrotor	Gripper-Only	Soft-Rigid	Y	Active	Passive	Below-Rod	Active
[50]	2025	Quadrotor	Gripper-Only	Rigid	Y	Active	Active	Below-Rod	Active
[14]	2024	FWAV	Gripper-Only	Soft	Y	Active	Passive	Above-Rod	Active
[25]	2024	Quadrotor	Gripper-Only	Soft	Y	Active	Active	Below-Rod	Active
[53]	2024	Quadrotor	Gripper-Only	Rigid	Y	Fully-Passive	Active	Below-Rod	Fully-Passive
[46]	2024	-	Leg-Gripper	Rigid	Y	Fully-Passive	Passive	Above-Rod	Active
[31]	2024	Quadrotor	Gripper-Only	Soft-Rigid	Y	Hybrid	Passive	Above-Rod	Active
[47]	2024	Quadrotor	Gripper-Only	Soft-Rigid	N	Fully-Passive	Active	Below-Rod	Hybrid
[24]	2024	Quadrotor	Leg-Gripper	Soft-Rigid	Y	Fully-Passive	Passive	Above-Rod	Passive
[29]	2023	Quadrotor	Gripper-Only	Soft-Rigid	Y	Fully-Passive	Passive	Above-Rod	Active
[32]	2023	Fixed-Wing UAV	Gripper-Only	Rigid	Y	Fully-Passive	Passive	Below-Rod	Hybrid
[13]	2022	FWAV	Leg-Gripper *	Soft-Rigid	N	Fully-Passive	Passive	Above-Rod	Active
[56]	2022	Quadrotor	Gripper-Only	Soft-Rigid	N	Fully-Passive	Passive	Below-Rod	Fully-Passive
[57]	2022	Quadrotor	Gripper-Only	Soft-Rigid	Y	Active	Passive	Below-Rod	Active
[33]	2021	Quadrotor	Leg-Gripper *	Soft-Rigid	Y	Passive	Passive	Above-Rod	Hybrid
[34]	2021	Quadrotor	Gripper-Only	Soft-Rigid	Y	Active	Active	Below-Rod	Active
[12]	2020	FWAV	Leg-Gripper	Soft-Rigid	Y	Active	Passive	Above-Rod	Active
[28]	2019	Quadrotor	Gripper-Only	Soft-Rigid	Y	Hybrid	Passive	Above-Rod	Active
[45]	2019	Hexa-copter	Leg-Gripper *	Soft-Rigid	Y	Fully-Passive	Passive	Above-Rod	Hybrid
[55]	2019	Quadrotor	Gripper-Only	Soft-Rigid	N	Fully-Passive	Passive	Below-Rod	Hybrid
[30]	2019	Quadrotor	Gripper-Only	Rigid	N	Active	Active	Below-Rod	Active
[48]	2018	Quadrotor	Gripper-Only	Soft-Rigid	Y	Active	Passive	Above-Rod	Active
[16]	2013	Quadrotor	Leg-Gripper	Soft-Rigid	Y	Fully-Passive	Passive	Above-Rod	Fully-Passive
[52]	2012	Quadrotor	Gripper-Only	Soft-Rigid	Y	Fully-Passive	Passive	Above-Rod	Active

* Actuated Leg; Y = Yes, N = No.

**Table 2:** Comparison of the examined grasping-based perching mechanisms. W = Perching Mechanism Weight; GF = Grasping Force; LC = Load Capacity; F/W = Force-to-Weight Ratio; SR = Perching Success Rate; $W_{UAV}$ = Total UAV Weight; MR = Mass Ratio; D = Perch Diameter Grasped.

Ref.	W [g]	GF [N] *	LC [N] *	F/W	CT [ms]	SR [%]	$W_{\mathrm{UAV}}$ [g] **	MR [%]	D [mm] ***
[26]	-	-	-	-	-	-	1500	-	30–110
[50]	390	-	269.8	70.5	-	-	2500	15.6	24–28
[15]	50 ^*a*^	16	-	32.6	-	87 ^*a*^	152 ^*a*^	32.9 ^*a*^	40–82
	100 ^*b*^	16	-	32.6	-	0 ^*b*^	550 ^*b*^	18.2 ^*b*^	40–82
[14]	39	-	6–9.5	15.8–25.0	-	100 ^*c*^	172	22.6	30–50
[24]	114	1–3.5	-	1.8–6.3	-	75	700	16.3	30–50
[47]	300	-	40	27.2	45	-	1800	16.7	20–55
[25]	55	0.8	-	1.5	-	-	304	18.0	-
[46]	89	-	39.1–76.1	45.0–87.6	-	95	-	-	10–40
[53]	-	0.9–3	-	-	-	-	-	-	85–200
[31]	142 ^*d*^	-	22 ^*d*^	15.8 ^*d*^	67 ^*d*^	-	950 ^*d*^	15.0 ^*d*^	54–103
	35 ^*e*^	-	10.7 ^*e*^	31.2 ^*e*^	42 ^*e*^	-	148 ^*e*^	23 ^*e*^	54–103
[32]	170	-	28	16.8	20	75	1020	16.7	40–55
[29]	-	-	12–176 ^*f*^	119.6 ^*f*^	4	80	-	-	55–11 ^*f*^
[13]	184	56.8	-	31.5	25	66	708	26.0	40–110
[56]	28	-	36.3	132	-	-	593	4.7	-
[33]	250	-	5.3	4.3	50	-	750	33.0	38–165
[34]	258	-	39.2	15.5	-	90	1950	13	-
[12]	-	-	-	-	-	-	450	-	-
[28]	551	-	56	10.4	96	-	-	-	48
[45]	178	-	-	-	-	-	-	-	24–184
[55]	9	-	-	-	-	-	36	25	-
[30]	-	-	-	-	-	-	1440	-	400
[48]	372	-	-	-	-	-	2300	16.0	60
[16]	478	-	-	-	-	73 ^*g*^; 98 ^*h*^	-	-	33–49
[52]	98	-	-	-	-	-	-	-	-

Legend: * All values refer to a single gripper and are given in Newtons for consistency. Where the original paper reported values in kilograms, these were converted assuming *g* = 9.81 m/s^2^. Ranges represent variations with perch size or geometry, while single values correspond to the specific test condition reported by the authors. ** Values include the perching mechanism’s weight. *** Values refer to diameters of cylindrical perches on which successful perching or grasping was demonstrated experimentally. Ranges indicate that multiple perch sizes were successfully grasped, while single values correspond to the only perch diameter tested. For consistency, in cases where the mechanism was tested on other geometries, only the cylindrical perch diameters are reported. - Value not available. ^*a*^ Value for entompoter; ^*b*^ value for ornithopter. ^*c*^ Success defined within a sufficiency region (≤1.1 m/s approach speed, ≤10 perch inclination). ^*d*^ Value for large gripper; ^*e*^ value for small gripper. ^*f*^ Values for three-finger grasper, taken as the reference configuration. ^*g*^ Drop; ^*h*^ Guided. Note: All values are approximate; some were taken from figures.

## 4. Discussion

Several perching mechanisms for aerial robots have been proposed over the last decade. Although not always specifically designed for flapping-wing platforms, they still offer valuable insights for developing effective solutions for different architectures.

Among these, ***leg-gripper*** mechanisms stand out as particularly suitable for larger flapping-wing robots, due to the specific needs associated with their landing maneuvers. As previously discussed, these robots are typically not VTOL-capable and approach the perch at relatively high forward speed. This results in the need for a perching mechanism to absorb the resulting impact energy to prevent damage to the robot architecture, both in terms of the structural skeleton and the kinematic system. A leg designed for this purpose can be beneficial. Moreover, as demonstrated by the perching mechanism developed by Zufferey et al. [13], an actuated leg can help compensate misalignments with the target perch, caused by oscillations induced by wing flapping. Such a leg could also contribute to allowing the robot to reach a stable equilibrium once perched. The introduction of additional weight is one of the drawbacks associated with integrating a robotic leg into an already complex system. Gripper-only mechanisms may be preferable for flapping wing robots with strict payload constraints, such as smaller ones. If these platforms possess hovering and associated VTOL capabilities, the need for an actuated leg mechanism is reduced, as shown by the two gripper-only systems successfully employed for the perching of such platforms by Broers et al. [14] and Hammad et al. [15].

The selection of appropriate ***materials*** for the gripper is fundamental. Designing a soft system [14,25] made entirely of compliant materials offers advantages such as low weight and inherent adaptability to several perch geometries. However, due to their limited gripping forces and structural stiffness, such designs are generally suitable only for lighter FWAVs, as the landing and grasping loads are relatively low. For heavier platforms, rigid or soft-rigid grippers are generally preferable. As can be observed in Table 1, these hybrid solutions are more common because they introduce a degree of flexibility that enhances adaptability to perch on surfaces without excessive loss in perching strength. Flexible materials are typically used in the joints connecting rigid segments [26,28] or as internal toe-pads [15,47]. These pads are sometimes covered with grip tape to help prevent slippage after landing, a critical aspect for non-hovering FWAVs, which could face difficulties in taking-off from an upside-down bat-like configuration if the gripper slips around the perch. A further solution to improve grip on natural surfaces, such as tree branches, involves the use of needles [46] or micro-spines [57], with the latter offering the capacity of adhering to rough surfaces with minimal pressing forces.

Regarding ***adaptability***, adaptive grippers, such as pre-stressed spring steel bands [29,31], underactuated designs with multi-phalange fingers [33,45,46,50] or Fin-Ray fingers [14,24] have demonstrated strong performance in grasping a wide range of perch geometries. Their ability to passively conform to irregular and varied shapes makes them particularly well-suited for use in natural environments, for instance, targeting real tree branches, and thus highly valuable for flapping-wing platform applications. On the other hand, non-adaptive grippers are more limited in the range of perch shapes they can securely anchor to. Therefore, these systems are less suitable for perching on natural branches, while remaining a good option in structured environments.

Another important characteristic that strongly influences the performance of perching mechanisms is the ***actuation method***. A well-established approach is the use of tendon-driven mechanisms, as demonstrated by the large number of existing designs that adopt this strategy. Those specifically developed for FWAVs [12,15] employ SMA springs as actuators. They exhibit a high power-to-weight ratio, but with modest energy efficiency and relatively slow response times (even if similar to bio-inspired examples, with bandwidth between 0.1 and 1 Hz), which limits their suitability for dynamic perching. Alternative solutions can be identified by examining tendon actuation strategies developed for the perching of multirotor and fixed-wing drones. A promising approach for achieving rapid, passive closure draws inspiration from the avian ADFM, leveraging gravity or energy deriving from the impact with the target pole or branch to collapse a leg architecture, which in turn pulls the gripper tendons and drives its closure. This strategy appears promising for larger FWAVs due to its lower impact on the system’s overall mass and the high available energy, while posing challenges for lightweight flapping-wing robots due to stricter payload constrains and the difficulty of generating sufficient grasping force from limited impact energy.

Depending on the configuration assumed by the robot after perching, these bio-inspired mechanisms can enable passive grasp maintenance under the robot’s weight. However, to ensure a firm hold against disturbances, integrating a locking system is recommended. Passive opening is typically also enabled by these mechanisms: once the locking mechanism, if present, is disengaged and the robot’s weight is removed during take-off, the legs return to their extended position, allowing the gripper to open. To assist and accelerate this process, elastic elements can be incorporated into the gripper to drive the finger open [33].

Another class of tendon-driven mechanisms achieves fast closure by leveraging the restoring forces of elastic joints. This approach has the additional advantage of enabling passive and secure grasp maintenance without the need for a locking system. For the same reasons discussed earlier, fully passive closing variants, such as the one proposed by Stewart et al. [32], are better suited to heavier, non-hovering ornithopters.

An alternative approach, scalable for both large and small FWAVs, is to introduce an active trigger to release the stored elastic energy, eliminating the need to rely on external events, such as impact with the target perch, to generate power. An example is proposed by [28], who introduced a servo to activate a quick-release mechanism. Typically, in mechanisms using this grasping strategy, an actuator is required to reopen the gripper by pulling the finger tendons and restoring the system’s elastic energy in preparation for the next grasp. This process may be time and energy intensive. A possible solution to enable passive and potentially faster reopening, as suggested by [32], is to design a mechanism that stores energy during the impact and later uses it for reopening.

Finally, fully-active tendon-driven grippers, in which servomotors are used to either pull [48] or release [26] the tendons, have also been proposed and represent a viable approach. However, due to the need to limit both energy consumption and actuator size, these systems often suffer from slower actuation speeds. This can be a limiting factor in fast perching scenarios, making such systems more suitable for FWAVs capable of vertical landings, which can approach the perch more slowly. These considerations also apply to other types of grippers actuated by electric motors, whether through mechanisms that allow simultaneous motion of all fingers [14,34] or by using individual servos controlling each finger separately [30]. In all these systems, as in other active designs, the same actuator is typically used for both closing and opening the fingers, helping to avoid the added weight and complexity of a separate reopening mechanism.

Another promising strategy for actuation in perching mechanisms for flapping-wing robots is fostering bistability. As demonstrated by the system proposed by Zufferey et al. [13], which was successfully used to enable perching for a large-scale ornithopter, a suitably designed bistable mechanism can enable a rapid, strong, and passive grasp, automatically triggered upon contact with the perch and maintaining a firm hold without requiring energy input. Among these devices, spring-steel bands stand out for fast closure, intrinsic adaptability, fatigue life, and favorable force-to-weight ratio, making them a feasible solution across the full range of FWAVs. However, the success of such grippers could be compromised by undesired bouncing at impact, as shown by Nguyen et al. [29]. Appropriate measures should be taken to mitigate this issue, such as introducing an active trigger [31]. Additionally, further testing is needed to validate the effectiveness of these mechanisms for stable perching from non-vertical approaches, which are typical of non-hovering FWAVs. Another drawback associated with this kind of architecture is the need for an actuator system to return it to the open state. Such a process is typically slow, adds further weight to the overall system and affects power consumption.

Moving to the soft-robotics Fin-Ray-based grippers, the system developed by Broers et al. [14] demonstrates that such designs can be effectively used for the perching of FWAVs capable of vertical landing. Additional examples developed for multirotor platforms suggest potential alternative actuation strategies for these grippers. Fully passive mechanisms proposed by Askari et al. [24], which leverage the platform’s weight acting on a Hoberman linkage, and by Lee et al. [53], which exploit the load acting on spring-loaded prismatic shafts to achieve closure, offer potential solutions to eliminate the need for actuators. These approaches may be suitable for hovering FWAVs, although their ability to generate sufficient gripping force, particularly for lightweight platforms, requires further investigation. Their applicability to non-hovering flapping-wing robots is also worth exploring. While the impact at landing could potentially be exploited to trigger a rapid closure, the approach angles typical of these platforms limit the possibility of continuing to apply force to the mechanism once the motion stops. As a result, without a locking mechanism, the gripper may reopen.

Finally, the soft actuator design proposed by Ching et al. [25] may be an interesting solution for the perching of FWAVs with limited payload capacity thanks to its low weight and adaptability. However, the applicability of this type of architecture is significantly conditioned by the need for continuous energy input to keep the gripper closed. Indeed, designs that rely on active holding, such as the one just mentioned, or those using electric motors without locking mechanisms, or incorporating locks that continuously require power (e.g., electroadhesive clutches [47]), can limit perching duration due to their ongoing energy demand. To enable prolonged perching, the energy consumption of the perching mechanism should be minimized. For this reason, a passive holding mechanism is generally preferable. As previously discussed, bistable structures, tendon-driven mechanisms that close under the restoring forces of their joints, or systems that close under the action of the robot’s weight, offer the advantage of enabling passive grasp maintenance. Alternatively, in both passively and actively closing systems, locking mechanisms, such as ratchets inspired by the avian DTLM [14,33,46] or latches engaged automatically at landing [32,56] or controlled by servos [45], can be integrated to ensure grasp maintenance over time without power consumption.

## 5. Conclusions

In conclusion, this review focused on grasping-based perching mechanisms for aerial robots, with particular attention to their application in flapping-wing platforms. Enabling FWAVs to perch on branches or other graspable structures is a valuable feature that can significantly extend their capabilities, supporting their use in a wide range of real-word applications.

A classification framework for these mechanisms was introduced with categories defined based on main system characteristics. Depending on whether or not the mechanism includes a leg, systems were divided into leg-gripper mechanisms and gripper-only designs. According to material stiffness, grippers were categorized as soft, rigid or soft-rigid. Closing strategies were used to classify systems as fully-passive, hybrid or active. For grasp maintenance, mechanisms were grouped into passive and active holding systems. Based on perching configuration, mechanisms were divided into above-rod and below-rod. Finally, according to opening strategies, systems were classified as fully-passive, hybrid or active. This classification served as a foundation for analyzing and summarizing existing designs developed over the years, first for flapping-wing platforms and then for other aerial vehicles such as multirotors and fixed-wing drones, which were included as valuable references for the development new perching mechanisms for flapping wing-robots. The review then discussed the advantages and limitations of the various proposed strategies, along with their suitability to flapping-wing robots, taking into account differences among these platforms in terms of payload capacity and landing behavior.

Through this analysis, the review aims to support the development of new designs that, in complement to those already proposed, can further enhance the practical usability of flapping-wing robots. Equipping FWAVs with perching capabilities opens a wide range of possibilities, such as enabling wildlife observation in natural environments or vehicle monitoring in urban areas with minimal disturbance to the population. Perching mechanisms could also make flapping-wing robots valuable tools for monitoring and inspecting infrastructures such as bridges, where the ability to perch on structural elements would facilitate repeated and prolonged data acquisition. Another promising application is the integration of FWAVs within the framework of Connected, Cooperative, and Automated Mobility (CCAM) in urban contexts, enabling a wide range of functions, including public service support and delivery.

Despite the progress made in recent years, several critical challenges remain before such tasks can be accomplished. To date, perching mechanisms for flapping-wing robots have been tested almost exclusively in structured laboratory environments under controlled conditions. Demonstrating reliable performance in real-world scenarios therefore represents a fundamental milestone that still needs to be achieved.

For non-hovering FWAVs, an important open challenge remains, achieving take-off after perching, as to the best of the authors’ knowledge, no large-scale ornithopter has yet demonstrated untethered take-off from a perch. The main difficulty lies in enabling the robot to leave the perch in a way that allows it to gain forward velocity while losing altitude, until the minimum speed required for sustained flight is reached. A possible pathway could involve developing perching mechanisms capable of providing an initial forward push to the robot. For FWAVs capable of vertical take-off, instead, this phase is generally less critical, as they can start generating lift before detaching from the perch and then open the gripper once the lift force is sufficient to sustain the vehicle’s weight and take-off. For these VTOL-capable platforms, future research could also explore perching on planar surfaces, further expanding their operational versatility.

Additional research directions specifically targeted to non-hovering FWAVs include exploring strategies that enable them to approach a perch with near-vertical trajectories by operating under full-stall conditions. Finally, a promising but largely unexplored application is the use of perching mechanisms for the autonomous grasping, transporting and releasing of objects.

## Data Availability

Data Availability Statement: No new data were created or analyzed in this study.

## Abbreviations

The following abbreviations are used in this manuscript:

ADFM	Automatic Digital Flexor Mechanism
APM	Automatic Perching Mechanism
DC	Direct Current
DTLM	Digital Tendon-Locking Mechanism
FiBa	Film-Balloon
FWAVs	Flapping-Wing Aerial Vehicles
PLA	Polylactic Acid
SMA	Shape Memory Alloys
TPU	Thermoplastic Polyurethane
UAVs	Unmanned Aerial Vehicles
VTOL	Vertical Take-Off and Landing
CCAM	Connected, Cooperative, and Automated Mobility
